# Ecological and Health Risk Assessments of Heavy Metals Contained in Sediments of Polish Dam Reservoirs

**DOI:** 10.3390/ijerph20010324

**Published:** 2022-12-25

**Authors:** Mariusz Sojka, Mariusz Ptak, Joanna Jaskuła, Vlerë Krasniqi

**Affiliations:** 1 Department of Land Improvement, Environmental Development and Spatial Management, Poznań University of Life Sciences, Piątkowska 94E, 60-649 Poznań, Poland; 2 Department of Hydrology and Water Management, Adam Mickiewicz University, Krygowskiego 10, 61-680 Poznań, Poland; 3 Department of Environmental Engineering, Faculty of Civil Engineering, University of Prishtina “Hasan Prishtina”, Agim Ramadani St., 10000 Prishtinë, Kosovo

**Keywords:** reservoir, sediments, heavy metals, ecological risk, health risk

## Abstract

This study aimed at investigating the distribution of heavy metals (HMs: Zn, Pb, Cd, Ni, Cr, and Cu) in the bottom sediments of 28 reservoirs covered area of Poland. The paper evaluates the pollution of sediments with HMs and their potential toxic effects on aquatic organisms and human health on the basis of results provided by the Chief Inspectorate of Environmental Protection in Poland. The average concentrations of HMs in the bottom sediments of the reservoirs were as follows: Cd < Ni < Cr < Cu < Pb < Zn. (0.187, 7.30, 7.74, 10.62, 12.47, and 52.67 mg∙dm^−3^). The pollution load index values were from 0.05 to 2.45. They indicate contamination of the bottom sediments in seven reservoirs. The contamination-factor values suggest pollution with individual HMs in 19 reservoirs, primarily Cr, Ni, Cu, and Pb. The analysis showed that only two reservoirs had the potential for toxic effects on aquatic organisms due to high concentrations of Cd and Pb. The hazard index values for all the analyzed HMs were less than one. Therefore, there was no non-carcinogenic risk for dredging workers. The reservoirs were divided into two groups in terms of composition and concentration values. Reservoirs with higher concentrations of HMs in bottom sediments are dispersed, suggesting local pollution sources. For the second group of reservoirs, HMs’ concentrations may be determined by regional pollution sources. The analysis showed that Pb, Zn, and Cd concentrations are higher in older reservoirs and those with higher proportions of artificial areas in their catchments. Concentrations of Ni, Cu, and Cr are higher in reservoirs in south Poland and those with higher Schindler’s ratios.

## 1. Introduction

Retention reservoirs exist all over the globe. Their basic function is the provision of water to meet municipal, industrial, and agricultural needs [1]. They are built to protect people against floods and droughts [2]. Moreover, they are used for fisheries (primarily fish farming) and recreational purposes [3]. Many of them also fulfill hydropower functions [4,5]. Approximately 4200 artificial reservoirs have been built in Poland. Only 100 of them have a volume greater than 1 million m^3^. Irrespective of their functions, the construction of a retention reservoir results in the modification of river flows, debris transport, and chemical compounds [6,7,8]. Reservoirs can serve as sources or sinks for heavy metals [9]. Changes in flow conditions result in debris sedimentation [10,11]. The type of debris deposited in the reservoir depends on the type of landscape (lowland, upland, mountains), the location of the reservoir in the catchment, and its geological structure. Debris is the primary carrier responsible for the supply of HMs to reservoirs [12]. The content of HMs in the aquatic environment is the subject of numerous studies [13,14,15,16,17] indicating the complexity of this issue. Sediment pollution is related to human activity [18]. HMs supplied to surface waters due to industrial activity, mining, municipal management, and agriculture are particularly dangerous [19,20,21]. Human activity contributes to an increase in the quantity of HMs supplied to sediments and affects their spatial distribution in the reservoir [22]. The transport of heavy metals is related to fine-grained debris (dust and loam) and organic matter [23]. The volume of sediment supply and composition of debris particles modulate the spatial distribution of metals in reservoirs [24]. Metals accumulated in reservoirs can be later released from the bottom sediments to the water column [25,26,27]. Heavy metals in excessive concentrations can have a toxic effect on aquatic organisms [28,29]. They can also be accumulated in tissues of such organisms, e.g., fish, and pose a threat to people consuming them [30,31,32]. The process of deposition of bottom sediment transported by a river over multiple years results in a decrease in the volume of reservoirs [33]. According to the report of the World Commission on Dams [34], a volume of 0.5 up to 1% is lost annually due to the sedimentation process. Therefore, periodical dredging works or other methods are applied, aimed at the restoration of the volume of sediments [35,36,37]. Deepening works and storage of sediments removed from the reservoir are very costly [38]. During dredging works, workers come in direct contact with sediments [39]. There is therefore a group of people potentially exposed to their toxic properties and further threatened with the occurrence of various diseases, in special cases including cancer [40]. The analysis of the quality of bottom sediments of retention reservoirs is of key importance from the point of view of the assessment of their potential toxic effect on aquatic organisms and human health [41,42,43,44]. Many indices have been developed that permit the assessment of the pollution of bottom sediments, and its potential toxic effects on aquatic organisms and humans [45,46,47]. The pollution of sediments determines the selection of the method for their future management [48]. Recycling management of waste materials is becoming increasingly popular around the globe. In this context, research focuses on finding solutions for the reuse and recycling of bottom sediments [49]. Sediments from reservoirs are used as fertilizers or agents to improve soil properties [50]. The accumulated heavy metals can be subject to sorption by plants [51]. Bottom sediments can be used as substrates for the growth of non-edible plants [52].

The objective of the paper was the assessment of the spatial variability of HM concentrations in the bottom sediments of retention reservoirs. The analysis was based on the example of 28 retention reservoirs in Poland. The study involved the determination of the degree of pollution of bottom sediments with HMs and the potential toxic effect of HMs on aquatic organisms and the health of workers conducting dredging works. Additionally, statistical methods were used to determine the sources of HMs. The analyzed reservoirs differ in terms of the operation period, surface area, volume, location within catchment, and human pressure due to activity conducted in catchments

## 2. Materials and Methods

### 2.1. Study Site Description

**Figure 1:**
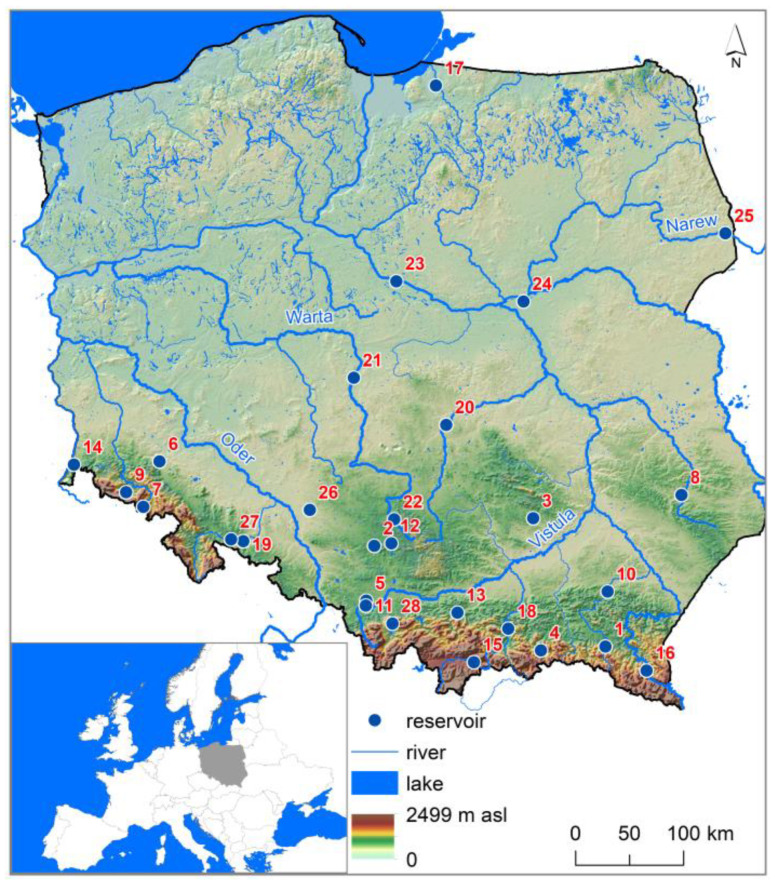
Reservoir location. Numbering of reservoirs in accordance with Table 1.

A total of 28 dam reservoirs in Poland were selected for the study (Figure 1). The Niedów Reservoir is located furthest to the west (No. 14), Siemianówka to the east (No. 25), Solina to the south (No. 16), and Pierzchały to the north (No. 17). The highest number of reservoirs is in the south of Poland in upland and mountainous areas. In terms of volume, the Soliński Reservoir is the largest (No. 16). The Pierzchały Reservoir (No. 17) has been operating since 1916, and the Nielisz Reservoir started operation in 2008 (No. 8) (Table 1). The largest reservoirs in terms of surface area are Włocławek (No. 23) on the Vistula River and Jeziorsko (No. 21) on the Warta River. Both reservoirs are located in central Poland in lowland areas. The Włocławek Reservoir also has the highest volume, reaching 408 × 10^6^ m^3^. The highest dam is constructed on the Solina Reservoir on the San River. It has a height of 60 m.

In terms of the water exchange period, the analyzed objects can be separated into rheolimnic, transitional, and limnic reservoirs [54]. Rheolimnic reservoirs are those where the water retention period is shorter than 20 days. In transitional reservoirs, the retention period is from 20 to 40 days, and in limnic reservoirs, the retention period exceeds 40 days. The analyzed reservoirs represent all these types. The shortest retention time was observed in the Wisłok Reservoir (No. 10), where it is 0.6 days and the longest is in Kozłowa Góra (No. 2)—which is 307 days [54]. The retention time affects the possibility of debris sedimentation in reservoirs, and therefore, retention of pollutants.

### 2.2. Materials

We employed data on HM concentrations in sediments of reservoirs provided by the Chief Inspectorate of Environmental Protection. The data were collected as part of the national environmental monitoring program carried out in 2018, 2019, and 2020. The study materials were the results of HMs analysis in the 5 cm surface layer of sediments. Sediments for the analysis were generally sampled near the dam. The number of sediment samples depended on the surface area of the reservoir. The following numbers of samples were collected from dam reservoirs depending on their areas: for reservoirs smaller than 2.5 km^2^ (1 sample), 2.5 to 5.0 km^2^ (2 samples), 5.1 to 10.0 km^2^ (3 samples), 10.1 to 50 km^2^ (4 samples), and larger than 50 km^2^ (5 samples). The bottom sediment sampling was performed according to the methodology specified in the PN-ISO 4364:2005 standard [55]. Elements selected for the analysis included Cd, Cr, Cu, Ni, Pb, and Zn. HM concentrations in sediment samples were determined utilizing inductively coupled plasma–optical emission spectrophotometry (ICP-OES). The extraction of sediment samples (grain fraction < 0.2 mm) was conducted using aqua regia. Limits of detection for Cd, Cr, Cu, Ni, Pb, and Zn were 0.05, 0.30, 0.40, 0.40, 1.0, and 0.5 mg·kg^−1^, respectively. Moreover, the paper employed data regarding the pH, electrolytic conductivity (EC), and total organic carbon in sediments (TOC).

### 2.3. Methods

The assessment of sediment pollution involved the calculation of the contamination factor (CF) [56] and pollution load index (PLI) [57]. The contamination factor [56] permits the calculation of pollution of sediments in reference to an individual element and is calculated from the following formula:

(1) $$CF_{i}=\frac{C_{i}}{B_{i}}$$

where *C_i_* is the measured concentration of metal *I* and *B_i_* is the geochemical background value of metal *i*. The following geochemical background values were adopted in this study: Cd—0.5 mg·kg^−1^, Cu—6 mg·kg^−1^, Cr—5 mg·kg^−1^, Ni—5 mg·kg^−1^, Pb—10 mg·kg^−1^, and Zn—48 mg·kg^−1^ [58].

Following [59], four contamination categories were distinguished: low contamination—CF < 1, moderate contamination −1 ≤ CF < 3, considerable contamination 3 ≤ CF < 6, and very high contamination—CF ≥ 6.

The pollution load index (PLI) [49] permits the calculation of accumulated pollution of sediments with all the analyzed elements and is calculated from the following formula:

(2) $$PLI=\sqrt[{n}]{\overset{n}{\underset{i=1}{\prod }}CF_{i}}$$

where *CF_i_* is the contamination factor value for i-th metal, and *n* is the number of heavy metals. Following, two contamination categories were distinguished: no pollution—PLI ≤ 1 and pollution—PLI > 1.

An analogical procedure was adopted for the analysis of the potential toxic effect of HMs accumulated in bottom sediments on aquatic organisms. The assessment of ecotoxicological impacts was carried out based on the potential ecological risk index of a single metal (ER_i_). In a complex context, the potential ecological risk index (PERI) was calculated [59]. The potential ecological risk (ER) index of a single metal [59] was calculated from the following formula:

(3) $$ER_{i}=TR_{i}\;\cdot \;\frac{C_{i}}{B_{i}}$$

where: $TR_{i}$ is the toxic response factor for metal *i*. The toxic response factors for Zn, Cr, Cu, Pb, Ni, and Cd were adopted according to Hakanson [58] with the following values: 1, 2, 5, 5, 6, and 30. Moreover, five categories of ecological risk were distinguished: low—$ER_{i}$ < 40, moderate—40 ≤ $ER_{i}$ < 80, considerable—80 ≤ $ER_{i}$ < 160, high— 160 ≤ $ER_{i}$ < 320, and very high $ER_{i}$ ≥ 320.

The Potential ecological risk index (PERI) [59] was calculated for the purpose of analyzing the accumulated toxic effect of the studied HMs on aquatic organisms from the following formula:

(4) $$PERI=\overset{n}{\underset{i=1}{\sum }}ER_{i}$$

Following Hakanson [59], four potential ecological risk categories were designated: low—PERI < 150, moderate—150 ≤ PERI < 300, high—300 ≤ PERI < 600, and very high—PERI ≥ 600.

Finally, the potential effects of metals on human health were assessed. The group most exposed to potential toxic impacts are workers conducting maintenance activities on reservoirs associated with sediment dredging [39]. According to the U.S. Environmental Protection Agency (US EPA), the assessment considers the type and magnitude of potential exposure to HMs [60,61,62,63]. There are three potential exposure pathways: contaminants ingestion, dermal contact, and inhalation. In the case of workers conducting dredging works on reservoirs, direct dermal contact or potential ingestion of sediments remaining on hands during meals is the most probable. This study, therefore, takes into consideration two exposure pathways, namely, ingestion and dermal contact. The average daily dose (ADD) of HMs in sediments through ingestion (ADD_ing_) and dermal contact (ADD_derm_) were calculated according to the following formulas:

(5) $$ADD_{ing}=C_{i}\frac{IngR\cdot EF\cdot ED\cdot CF}{BW\cdot AT}\;\left(\mathrm{mg}\cdot {\mathrm{kg}}^{-1}\cdot {\mathrm{day}}^{-1}\right)$$

(6) $$ADD_{derm}=C_{i}\frac{SA\cdot AF\cdot ABF\cdot EF\cdot ED\cdot CF}{BW\cdot AT}\;\left(\mathrm{mg}\cdot {\mathrm{kg}}^{-1}\cdot {\mathrm{day}}^{-1}\right)$$

where *C_i_* is the measured concentration of metal *i*. The definitions of all other parameters and the values adopted for the calculations of the daily exposure dose of HMs through ingestion and dermal contact are provided in Table 2. The US EPA methodology was used for the calculations [60,61,62,63]. The time of exposure of workers was adopted with the assumption that dredging works will be conducted for 180 days in a year and the exposure period will be 35 years. The skin surface area parameter based on data from US EPA [63] assumes contact through hands and forearms. Moreover, the skin adherence factor was adopted as for irrigation installers.

The hazard quotient (HQ) was used to estimate the non-carcinogenic effect of individual HMs in sediments [61].

(7) $$HQ=\frac{ADD}{RfD}$$

The HQ is the ratio of the ADD of a heavy metal to its reference dose (RfD) for the same exposure pathway [60]. The RfD is the maximum daily dose of HMs from a specific exposure pathway that is considered to pose no appreciable risk of adverse effects to persons over a lifetime. If ADD is less than or equal to RfD (HQ ≤ 1), it is considered that no adverse health effects will occur, and if ADD exceeds RfD, (HQ > 1), it is likely that adverse health effects will occur [60,61,62,63,64,65].

To assess the total risk of a non-carcinogenic element in the three exposure pathways for a single element, the hazard index (HI) is calculated as follows:

(8) $$HI=\sum_{i=1}^{n}HQ_{i}=\sum_{i=1}^{n}\frac{ADD}{RfD}$$

The preliminary statistical analysis of data first involved the calculation of values of basic statistics. This permitted the assessment of the variability of HMs concentrations, detection of outliers, and determination of distribution. The correlation analysis aimed at the determination of correlations between particular HMs in sediments, and therefore, preliminary identification of their sources. The appropriate statistical analysis aimed at grouping reservoirs by HMs content in bottom sediments. Hierarchical cluster analysis was applied for this purpose (CA). The analysis was conducted employing the Ward method, with square Euclidean distance as the similarity measure. To identify potential pathways and sources of HMs supply to sediments of retention reservoirs, principal component analysis was conducted (PCA). PCA was conducted for the following variables: reservoir location (Lon—longitude; Lat—latitude; Alt—altitude), reservoir parameters (Ag—age; A—area; V—volume; RT—retention time; SD—shoreline development; SR—Schindler’s ratio); catchment parameters (CA—catchment area), catchment land cover (Agr—agriculture area; Urb—urban area; Wet—wetland area, Wat—water area; For—forest area). The values of the dam reservoir and catchment parameters are presented in Table 1 and Table S1. Moreover, the correlation analysis employed readings of pH, EC, and TOC. The number of significant principal components was selected based on the Kaiser criterion of eigenvalues higher than 1. Additionally, it was assumed that when factor loadings between the concentrations of selected HMs and rare earth elements REEs and principal components are 0.75–1.00, 0.50–0.75, and 0.30–0.50, they are adequately strongly, moderately, and weakly correlated [66]. CA and PCA were conducted using Statistica 13.1.

## 3. Results

HM content in the bottom sediments of retention reservoirs can be arranged based on their average concentration in the following increasing order: Cd < Ni < Cr < Cu < Pb < Zn (Table 3). The mean Cd concentration in sediments was 0.19 mg∙kg⁻^1^. Cd concentrations were below 0.05 mg∙kg⁻^1^, i.e., below the limit of detection in the applied analytical method, in as many as 18 cases. In the remaining cases, Cd concentrations were at a level from 0.08 to 1.65 mg∙kg⁻^1^. Concentrations of Ni and Cr in the bottom sediments of the analyzed retention reservoirs were approximate. The mean Ni concentration reached 7.30, with minimum and maximum values of 0.20 and 32.20, respectively. The mean Cr concentration was 7.43. The minimum value was 0.53, and a maximum value was 31.7. Cu and Pb concentrations were somewhat higher, reaching 10.62 and 12.47, respectively.

**Figure 2:**
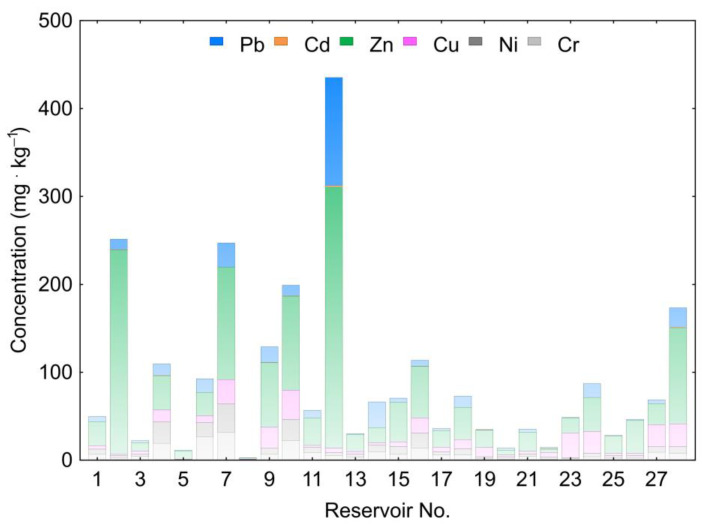
Cumulative concentrations of HMs in analyzed reservoirs.

It should be emphasized that the range of Cu concentrations in the reservoirs varied from 0.2 to 33.2 and was similar to those of Ni and Cr. More variability was recorded for Pb, which had concentrations from 0.5 to 123.0. The analysis of Ni, Cr, Cu, and Pb concentrations utilizing a Wilcoxon test showed no statistical significance for the differences in their content. It therefore cannot be stated that the concentration of one of these elements was higher than those of the remaining ones in the sediments of retention reservoirs in Poland. The highest concentrations in bottom sediments were recorded for Zn, averaging 52.67 and ranging from 1.1 to 297.0. The cumulative concentrations of heavy metals in individual reservoirs are presented in Figure 2.

**Figure 3:**
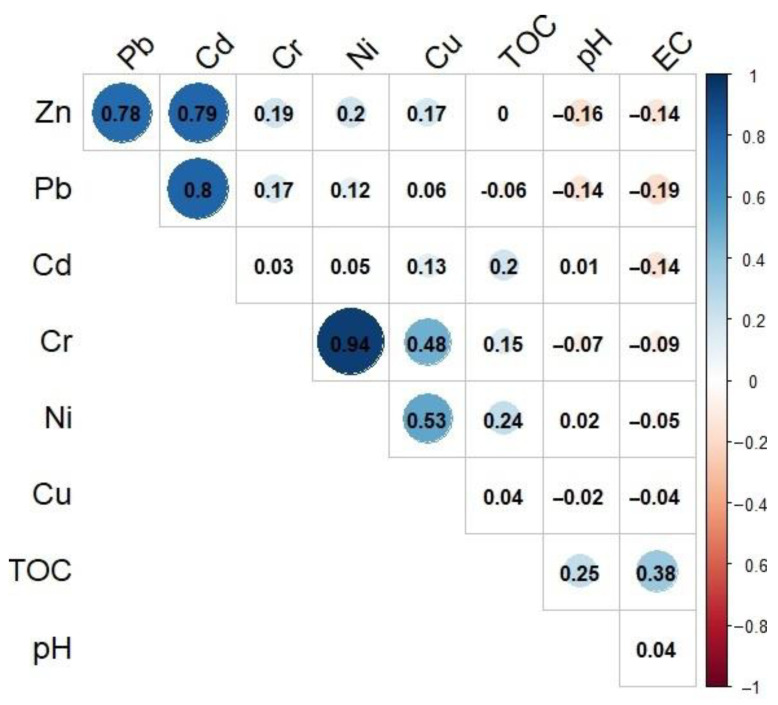
Results of correlation analysis.

In the case of each of the analyzed HMs, the concentrations were observed to show a positively skewed distribution. The analysis of HMs concentrations utilizing a Shapiro–Wilk test showed statistically significant differences in the distributions from normal distribution at a level of 0.05. The analysis of the dataset utilizing a Grubbs test in terms of the occurrence of outliers showed that no outliers occurred only for Cu concentrations. For Cd, Pb, and Zn, outliers occurred in the Przeczyce Reservoir (No. 12) and for Cr and Ni in the Bukówka Reservoir (No. 7). Moreover, the characteristic values of pH, electrical conductivity, and total organic carbon are presented in Table 3. The correlation analysis showed the strongest correlations with concentrations of Ni and Cr (0.94). This suggests a similar pathway of their supply to the reservoirs. The analyzed elements can be divided into two groups. The first one covers mutually correlated concentrations of Pb, Zn, and Cd (values of correlation coefficients from 0.78 to 0.80). In the second group of elements, including Cr, Ni, and Cu, correlations are significant at a level of 0.05, although their values show higher variability, from 0.48 to 0.94. The analysis found no correlations between the analyzed elements and pH, EC, and TOC in the sediments (Figure 3).

**Figure 4:**
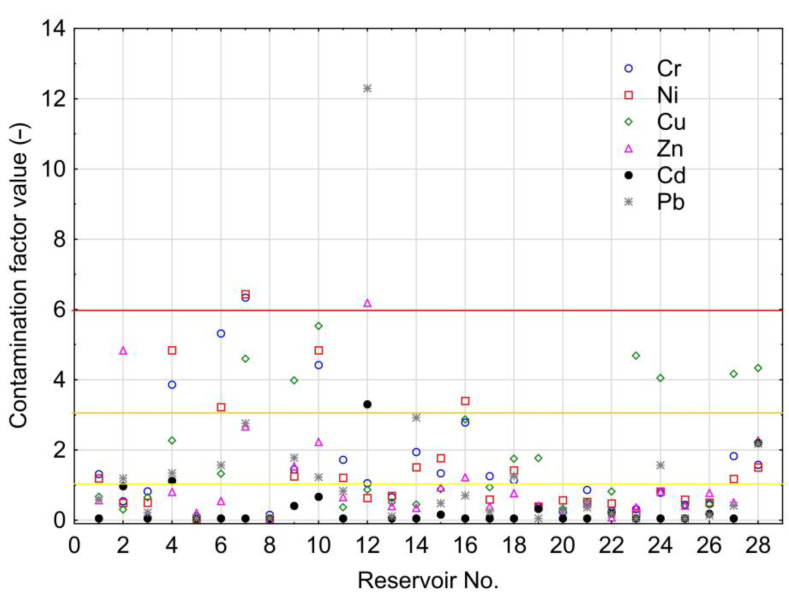
Values of contamination factors for the analyzed HMs in bottom sediments of retention reservoirs in Poland.

The analysis of pollution of bottom sediments in reference to individual HMs based on mean values of the contamination factor (CF) showed that their pollution can be arranged in the following increasing order: Cd < Zn < Pb < Ni < Cr < Cu. Results of pollution of bottom sediments with particular HMs are presented in Figure 4. CF values above one suggest pollution of bottom sediments. CF values above one were usually recorded for Cr (15 times), and most seldom for Cd (3 times). In four cases, CF values were higher than six, suggesting very high contamination.

**Figure 5:**
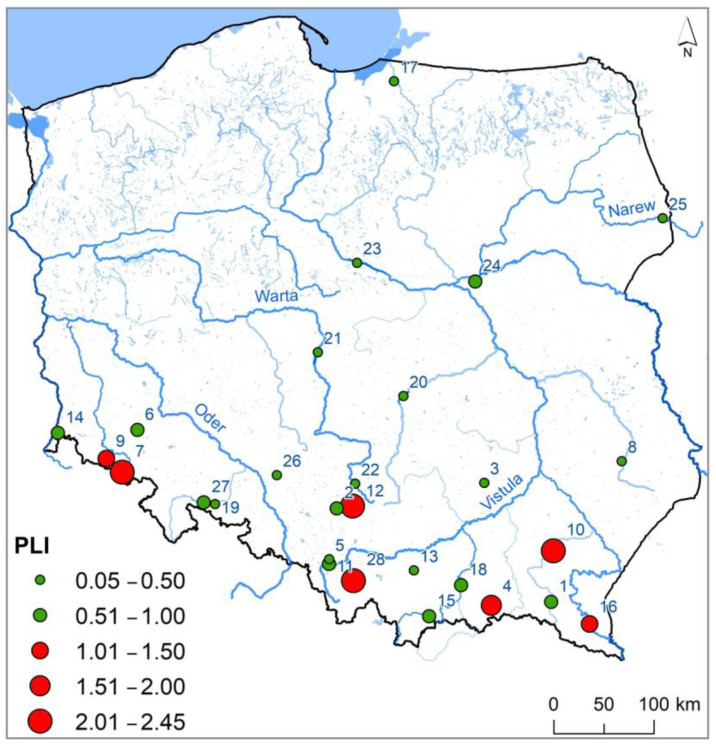
State of pollution of bottom sediments of retention reservoirs with HMs based on the values of the pollution load index.

In the bottom sediments of the Bukówka Reservoir (No. 7), very high contaminations of Cr and Ni occurred, and in the Przeczyce Reservoir (No. 12), there were very high contaminations of Zn and Pb (Figure 2). The analysis showed that in the Chańcza (No. 3), Łąka (No. 5), Nielisz (No. 8), Dobczyce (No. 13), Zarzęcin (No. 20), Jeziorsko (No. 21), Poraj (No. 22), Siemianówka (No. 25), and Turawa (No. 26) reservoirs, CF values for each HMs were lower than 1. This suggests a lack of pollution of sediments with HMs. Considering PLI values illustrating accumulated pollution of sediments with HMs, they were present at 0.05 to 2.45 mg∙kg^−1^ and were on average 0.82 mg∙kg^−1^. The highest value of the PLI index was recorded in the sediments of the Rzeszów Reservoir (No. 10), and the lowest for the Nielisz Reservoir (No. 8). In the bottom sediments of seven reservoirs, PLI values were higher than one (Figure 5—highlighted in red), suggesting pollution of bottom sediments with HMs. It should be emphasized, however, that in 12 reservoirs (among 21 where PLI values were lower than 1), based on CF values, pollution with individual HMs was determined. Figure 5 shows that the highest PLI values existed in reservoirs in the south of Poland, although their distribution is dispersed.

**Figure 6:**
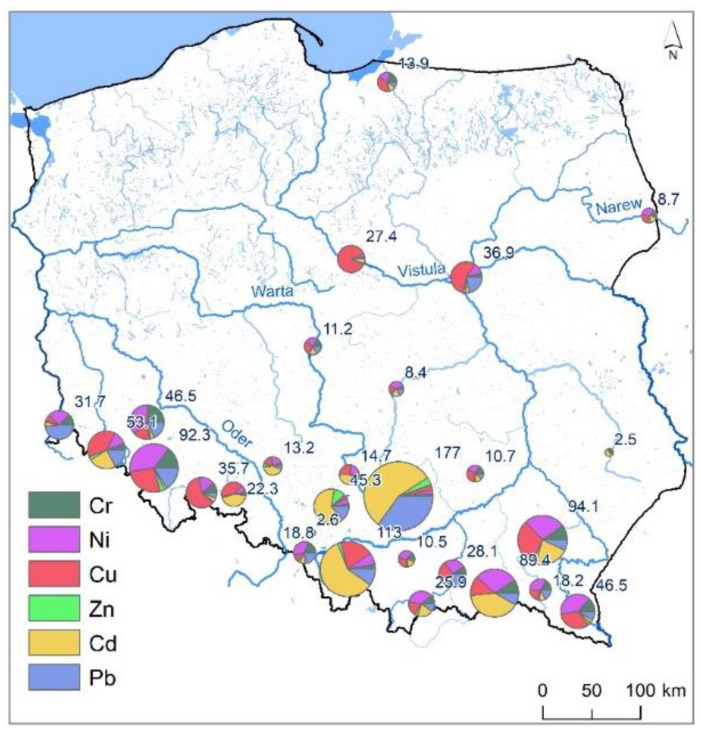
Values of the PERI index calculated in total for concentrations in bottom sediments of retention reservoirs in Poland.

The analysis of the potential toxic effect of individual HMs showed that the toxicity was low to considerable. ERi values varied from 0.02 to 99. Mean ERi values can be arranged in the following increasing order: Zn < Cr < Pb < Ni < Cu < Cd. ER values for Cd and Pb in the Przeczyce Reservoir (No. 12) point to considerable and moderate potential ecological risks, respectively. In the Międzybrodzie Reservoir (No. 28), ER values for Cd reached 66, which also points to a moderate potential ecological risk. In the remaining cases, ER values were lower than 40, suggesting low potential ecological risk. PERI values describing the accumulated toxic effect on aquatic organisms varied from 2.4 to 176.3, averaging 37.8. The assessment of the accumulated toxic effect of HMs contained in sediments based on the PERI index suggests moderate potential ecological risk in the case of the bottom sediments of the Przeczyce Reservoir (No. 12). In the remaining cases, PERI values were lower than 150 (low potential ecological risk). The spatial variability of the PERI index in the bottom sediments of the analyzed retention reservoirs is presented in Figure 6.

**Figure 7:**
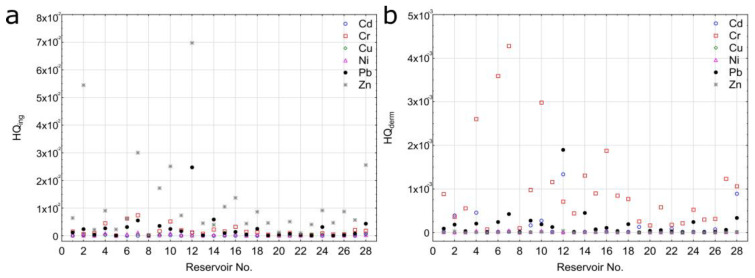
HQ values for the pathway related to ingestion (**a**) and dermal contact (**b**).

The assessment results of health risks due to HMs exposure in bottom sediments of Polish reservoirs are presented in Figure 7.

**Figure 8:**
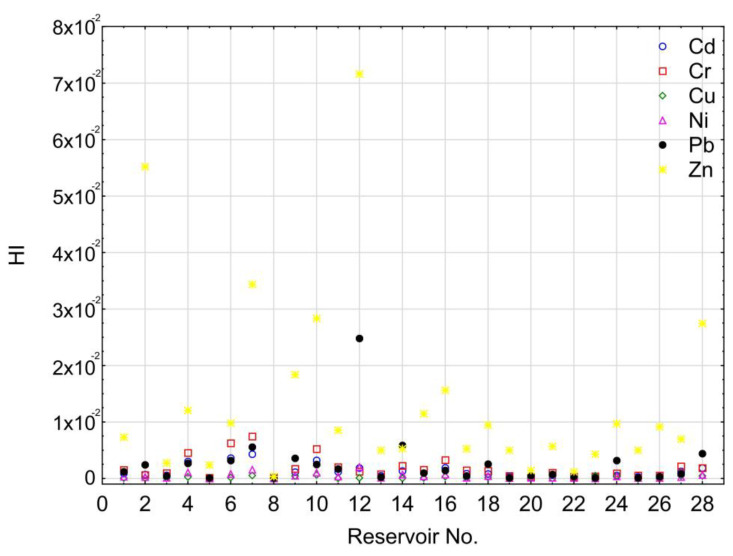
HI values point to a threat to workers conducting dredging works related to the removal of sediments from reservoirs.

Mean HQ values for the ingestion pathway for workers conducting maintenance work related to the removal of bottom sediments from the reservoir for Cr, Cd, Pb, Zn, Cu, and Ni were 1.82 × 10^−3^; 1.31 × 10^−4^; 2.51 × 10^−3^; 1.24 × 10^−2^; 1.87 × 10^−4^; and 2.57 × 10^−4^, respectively. HQ values for the dermal pathway for Cr, Cd, Pb, Zn, Cu, and Ni were 1.05 × 10^−3^; 1.51 × 10^−4^; 1.92 × 10^−4^; 7.11 × 10^−6^; 7.17 × 10^−6^; and 1.10 × 10^−5^, respectively. The results suggest a considerably greater risk in reference to Pb, Ni, Cu, and Zn concerning the ingestion pathway. HQing values were on average higher than HQderm values 13, 24, 26, and 1740 times in reference to Pb, Ni, Cu, and Zn, respectively. In the case of Cr, HQing values were on average 1.7 times higher than HQderm values. For Cd, HQderm was on average 1.2 times higher than HQing. For individual elements, the share of HQ from ingestion in HI varied from 46.5% for Cd to 99.9% for Zn. In general, for Pb, Ni, Cu, and Zn, the share of HQing in HI exceeded 90% (92.9%, 95.9%, 96.3%, and 99.9%). This points to a dominant threat related to ingestion. This was also confirmed by other research [67,68]. The approximate effect of the ingestion and dermal pathways occurred for Cd and Cr. However, in only the case of Cd, a somewhat higher supply of the element to the organism is related to dermal contact. HI values for the analyzed reservoirs can be arranged in the following increasing order: Cu < Ni < Cd < Pb < Cr < Zn. HI values for all the analyzed HMs were lower than 1 due to no non-carcinogenic risk for dredging workers (Figure 8).

**Figure 9:**
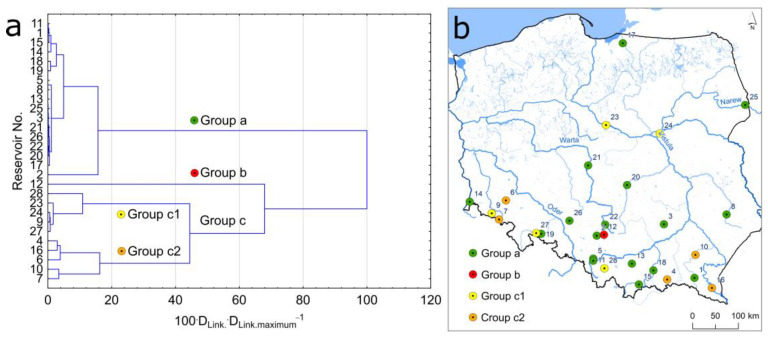
Probability of pollution of bottom sediments with HMs. Results of CA analysis (**a**) and spatial distribution of the designated groups (**b**).

The cluster analysis permitted the designation of three groups of reservoirs (Figure 9a). Group A included 17 reservoirs. In these reservoirs, Cr, Cu, Ni, Pb, and Zn concentrations were lower than values recorded in reservoirs included in Group C. The differences were statistically significant at a level of 0.05 based on a non-parametric U Mann–Whitney test. Group C covered 10 reservoirs. The variability of HMs concentrations in this group was high, resulting in the division of the group into two subgroups, C1 and C2. The subgroups included five reservoirs each. The analysis of the significance of differences in HM concentrations in both subgroups showed that Cr and Ni concentrations in subgroup C2 were higher than in subgroup C1. The differences were statistically significant at a level of 0.05. Group B included only Przeczyce Reservoir (No. 12), where the concentrations were very high. According to earlier analysis utilizing a Grubbs test of data in terms of the occurrence of outliers, in that reservoir, Cd, Pb, and Zn concentrations largely deviated from those recorded in other reservoirs. The spatial distribution of reservoirs included in particular groups based on HMs concentrations in bottom sediments is presented in Figure 9b. It should be emphasized that reservoirs where bottom sediments have higher HMs concentrations are dispersed throughout Poland. An exception may be a group of reservoirs located in the direct vicinity of Słup (No. 6), Bukówka (No. 7), and Sosnówka (No. 9). HMs concentrations in their sediments may be determined by similar factors with regional ranges. The distribution of the remaining reservoirs points to a more probable occurrence of local pollution.

**Figure 10:**
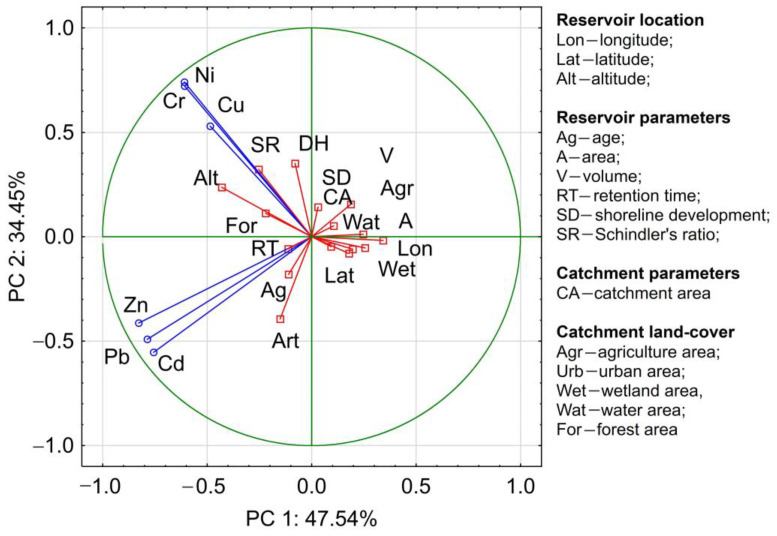
Dependency between HM content in bottom sediments of reservoirs and morphometric parameters of reservoirs and characteristics of catchments.

The principal component analysis permitted the designation of two groups of HMs. The first group included Ni, Cu, and Cr; and the second group, Pb, Zn, and Cd. Elements included in the second group occur in higher concentrations in the bottom sediments of reservoirs in catchments with larger shares of artificial land use. Moreover, higher concentrations of these elements occur in older reservoirs (Figure 10). The presence of elements included in the first group is higher in reservoirs located in upland and mountain areas, and those with higher Schindler’s ratios.

Although the PCA analysis does not indicate the impact of catchment land cover structure on sediment pollution, it was decided to analyze the land-use structure in the catchments of the most polluted and least polluted reservoirs. The most contaminated reservoirs according to PLI index values are Klimówka (No. 2), Bukówka, (No. 7), Sosnówka (No. 9), Rzeszów (No. 10) Przeczyce (No. 12), Solina (No. 16), and Międzybrodzie (No. 28). In these reservoir catchments, the ratio of urbanized areas ranges from 0.9 to 13.2%. However, when considering the entire set of catchments, the urbanized areas ranged from 0.7 to 15.4%. Other areas from which HMs can be delivered are agricultural areas. In the most contaminated catchments, the proportion of agricultural land ranges from 12.8 to 57.0%. On the other hand, in the whole set of catchments, the share of agricultural areas ranges from 12.8 to 69.4%. In addition, in the Klimówka (No. 2) and Solina (No. 16) reservoirs, there is a high proportion of forests, 86.7 and 84.2%, respectively, which should not affect the HM supply. The least contaminated bottom sediments were in the dam reservoirs Łąka (No. 5) and Nielisz (No. 8). In these reservoir catchments, the proportions of urbanized land were 15.4 and 4.3%, and those of agricultural land were 69.4% and 60.2%, respectively. These results indicate that the main impact can be attributed to local sources and that the supply of HMs can be caused by point pollution sources.

## 4. Discussion

The construction of dam reservoirs contributes to the disturbance of the continuity of a river. As a result, debris transport is inhibited, and debris accumulates in the reservoir, along with pollutants. Rivers transport potentially toxic pollutants [69,70,71]. Among these pollutants, heavy metals are investigated the most frequently (Table 4) due to their negative effects on people, animals, and plants.

The process of debris and pollutant sedimentation in a reservoir is complex and depends on the volume of the inflow to the reservoir; the parameters of the reservoir, the size, character, and location of pollution sources; and the pattern of operation of the reservoir [23]. The results of a study by Zhao et al. [9] confirm that heavy metal concentrations in sediments vary spatially and temporally. The surface layers of sediments analyzed in this article show the current situation in the catchment. Further layers of bottom sediments and the concentration of different elements in them offers a historical record of the situation occurring in the catchment [85,86]. As shown by the conducted analyses, it is very difficult to precisely identify the sources of origin of particular heavy metals in the bottom sediments of retention reservoirs due to the overlapping effect of several pollution sources, both local and regional. Research by other authors also evidences the overlapping effect of sources of different pollutants responsible for the supply of particular elements [87,88,89,90]. The character of the pollution source determines the further toxic effect of individual elements [91]. The study showed that higher HMs concentrations occur in older reservoirs [11]. The results correspond with those obtained by Bing et al. [22] which also evidenced that pollution of sediments with trace elements increased with time. Moreover, the content of pollutants increases with increases in the proportion of artificial areas and reservoirs with large catchment areas. Research by Sojka et al. [72] also showed that reservoirs with more frequent water exchange have higher concentrations of HMs. Geochemical research on bottom sediments shows that the highest accumulation of HMs occurs in the upper part of the reservoir and at the dam embankment, and the lowest in the middle part of the reservoir [81]. The diagram of water-sediment regulation affects the redistribution and accumulation of heavy metals in sediments [92]. Therefore, results obtained in this study based on samples collected at the dam generally showed the highest concentrations. Answering the question, however, requires expanding the monitoring network to cover all parts of reservoirs. The accumulation of sediments in reservoirs leads to their shallowing [93,94]. Due to this, works are periodically conducted aimed at their removal from the reservoir. Knowledge of pollution of bottom sediments is necessary to plan works related to their removal to avoid secondary pollution [28,95]. Pollutants deposited in bottom sediments can also have a negative effect on dredging workers who come in direct contact with such sediments. In the reservoirs analyzed in this paper, hazard index values for all the analyzed HMs were lower than one, suggesting a carcinogenic risk for dredging workers. Other researchers also analyzed the effect of pollution of sediments in this context. The research revealed a potential carcinogenic risk in reference to individual elements [96]. Moreover, the content of pollutants in bottom sediments determines the methods of their management. Monitoring research on retention reservoirs in only one place should be considered preliminary. Its objective is to identify objects among the entire population that should be subject to detailed analysis in the future. The analysis should cover detailed identification of the existing and historical pollution sources. Moreover, on reservoirs with higher-than-average pollution, the monitoring network and research should be expanded to cover the entire reservoir. The analysis of the potential accumulation of pollutants in aquatic organisms should also be performed, particularly those consumed by people. Geochemical research should be accompanied by ecotoxicological monitoring. Similar conclusions were drawn by Baran et al. [84].

Dominant sources of HMs in sediments differ at the continental scale. In North America, the main source is mining and industry; in Africa it is bedrock weathering; and in Europe it is mainly connected with wastewaters [26]. According to results obtained by Sojka et al. [26], HM contamination can be classified in the order Cd < Ni < Cr < Cu < Pb < Zn for 77 lakes located in the northern part of Poland. The results obtained for reservoirs analyzed in this study show the same order of HMs, Cd < Ni < Cr < Cu < Pb < Zn. According to Nawrot et al. [97] Zn, Pb, Cu, Cd, Ni, Ce, and As were mostly observed in urban and catchments. In this study, it was observed that Pb, Zn, and Cd concentrations were higher in older reservoirs located in the catchment with a greater proportion of artificial areas. Results obtained by Sojka et al. [26] showed that HMs can have toxic effects on aquatic biota only in single lakes mainly due to concentrations of Cu, Cd, and Pb that were higher than PEC values. Similar results were obtained in this study, EP values showed a potentially toxic effect on aquatic organisms due to high Cd and Pb concentrations only in two reservoirs. The results obtained by Hahn et al. [98] showed that main source of heavy metals in Klingenberg Reservoir in Germany is connected with soils in catchments. Sediments were mainly contaminated with Cd and Zn and released from soils by leaching processes. Additionally, the concentrations of As and Pb in a reservoir were influenced by soil erosion and by anthropogenic sources in the catchment. Buccione et al. [99] analyzed HM concentrations in Pietra del Pertusillo reservoir in Italy and indicated that contamination by Cr, Cu, Zn, As, and Pb is mostly driven by geogenic processes. A high concentration of As is considered as the most relevant for the environment and health, is highly toxic in its inorganic form, and can have immediate toxic effects for human health.

## 5. Conclusions

The analyses lead to the following detailed conclusions:

1. Pollution load index values suggest that bottom sediments in seven reservoirs were polluted. Moreover, in the other 12 reservoirs, contamination factor values point to pollution with individual HMs, primarily Cr, Ni, Cu, and Pb.
2. Ecological risk (ER) index values showed a potentially toxic effect on aquatic organisms due to high Cd and Pb concentrations only in two reservoirs.
3. Hazard index values for all the analyzed HMs were lower than one due to no carcinogenic risk for dredging workers. It was evidenced that ingestion of HMs leads to a greater risk for dredging workers.
4. In terms of the distribution and magnitude of concentrations, the analyzed reservoirs can be divided into two groups. The exception is the Przeczyce Reservoir, having much higher Pb, Zn, and Cd values.
5. Reservoirs where bottom sediments showed higher HMs concentrations are dispersed, suggesting the dominant role of local sources. For the Słup, Bukówka, and Sosnówka Reservoirs, HM concentrations can be determined by factors with a regional range.
6. The analyzed HMs were divided into two groups, one including Pb, Zn, and Cd; and the other, Cr, Ni, and Cu. This points to the analogical sources of their supply and factors responsible for their transport and accumulation in bottom sediments.
7. The analysis showed that Pb, Zn, and Cd concentrations are higher in older reservoirs located in the catchment with a greater proportion of artificial areas. Ni, Cu, and Cr concentrations are higher in reservoirs located in the south of Poland and those with higher Schindler’s ratios.

## Figures and Tables

**Table 1:** Parameters of the analyzed retention reservoirs (own elaboration based on Statistical [53], Picińska-Fałtynowicz and Błachuta 2012 [54]).

No.	Reservoir	River	Operation Start	Area (km^2^)	Volume (10^6^ m^3^)	Dam Height (m)	Retention Time (d)
1	Besko	Wisłok	1978	1.3	14.2	38.0	60
2	Kozłowa Góra	Brynica	1938	5.8	15.8	7.0	307
3	Chańcza	Czarna Staszowska	1985	4.7	23.8	15.0	218
4	Klimkówka	Ropa	1994	3.1	43.5	37.7	148
5	Łąka	Przszczynka	1985	4.2	12	6.0	80
6	Słup	Nysa Szalona	1978	4.9	38.6	19.2	22
7	Bukówka	Bóbr	1987	2	16.8	22.0	194
8	Nielisz	Wieprz	2008	8.3	25.6	8.6	107
9	Sosnówka	Czerwonka	2001	1.8	14	30.0	162
10	Rzeszów	Wisłok	1974	0.42	0.84		0.6
11	Goczałkowice	Mała Wisła	1956	32	168.4	14.0	80
12	Przeczyce	Czarna Przemsza	1963	4.7	20.7	7.4	109
13	Dobczyce	Raba	1986	10.7	137.7	31.0	146
14	Niedów	Witka	1962	1.9	4.9	12.0	12.6
15	Czorsztyn	Dunajec	1997	12.3	231.9	54.5	116
16	Solina	San	1968	21.1	474	60.0	299
17	Pierzchały	Pasłęka	1916	2.4	11.5	10.0	8.5
18	Rożnów	Dunajec	1942	16	159.3	31.5	31
19	Nysa	Nysa Kłodzka	1971	20.7	124.7	13.3	59
20	Sulejów	Pilica	1973	23.8	84.3	11.3	38
21	Jeziorsko	Warta	1986	42.3	202.8	11.5	56
22	Poraj	Warta	1978	5.1	25.1	12.2	97
23	Włocławek	Wisła	1970	70.4	408	12.7	4.5
24	Dębe	Narew	1963	30.5	94.3	7.0	8.2
25	Siemianówka	Narew	1995	32.5	79.5	9.2	198
26	Turawa	Mała Panew	1948	20.8	106.2	13.6	115
27	Otmuchów	Nysa Kłodzka	1933	19.8	143	19.8	61
28	Międzybrodzie	Soła	1936	3.7	32	21.2	22

**Table 2:** Assumptions used to calculate average ingestion and dermal contact doses of metals for employees dredging sediments from reservoirs (US EPA 64).

Parameter	Definition	Value for Workers	Unit
IngR	Ingestion rate	100 *	mg·day^−1^
EF	Exposure frequency	180 *	days·years^−1^
ED	Exposure duration	35 *	years
CF	Conversion factor	10^−6^	kg·mg^−1^
BW	Body weight	70	kg
AT	Average time of exposure	12,775	days
SA	Skin surface area parameter	2300	cm^2^
AF	Skin adherence factor	0.5	mg·cm^−2^·day^−1^
ABF	Dermal absorption factor	0.001	-

*—Values adopted by the authors.

**Table 3:** Characteristic concentrations of heavy metals (mg kg^−1^), pH value, electric conductivity (EC in µS cm^−1^), and total organic carbon (TOC in %) in bottom sediments of dam reservoirs in Poland.

Statistics	Cd	Cr	Cu	Ni	Pb	Zn	pH	EC	TOC
Number	28	28	28	28	28	28	28	28	28
Minimum	0.025	0.53	0.20	0.20	0.50	1.11	6.50	36	0.5
Mean	0.187	7.74	10.62	7.30	12.47	52.67	7.84	126	7.3
Median	0.025	5.49	5.28	3.80	5.38	26.80	7.80	94	5.9
Maximum	1.650	31.70	33.20	32.20	123.0	297.0	8.80	564	30.4
Standard deviation	0.371	8.01	10.40	8.06	23.21	68.45	0.62	108	6.9

**Table 4:** Examples of average metal concentrations in selected reservoirs (mg/kg).

Reservoir Location	Cd	Cr	Cu	Ni	Pb	Zn	Reference
Jeżewo, Poland	0.4	6.5	10.1	5.9	17.6	903.7	Sojka et al. [72]
Jutrosin, Poland	0.2	3.1	3.6	3.7	6.2	23.1	Sojka et al. [72]
Pakosław, Poland	0.1	2.0	2.0	2.0	2.6	221.7	Sojka et al. [72]
Rydzyna, Poland	0.1	3.5	4.2	4.4	5.9	436.5	Sojka et al. [72]
Środa, Poland	0.2	4.8	4.6	3.5	7.4	357.5	Sojka et al. [72]
Września, Poland	0.4	6.1	9.4	5.5	15.2	678.4	Sojka et al. [72]
Jinzai, Japan	-	42.0	34.0	28.0	24.0	215.0	Ahmed et al. [73]
Rybnik, Poland	3.7	32.2	258.3	20.4	67.6	439.4	Baran et al. [74]
Three Gorges, China	1.0	86.4	49.5	38.6	54.5	185.1	Bing et al. [75]
Pelham, USA	-	46.0	51.0	-	19.0	86.0	Clark et al. [76]
Grand Anicut, India	-	139.4	39.9	7.7	5.4	85.6	Dhanakumar et al. [32]
Anaikarai, India	-	45.4	10.3	2.0	2.2	24.7	Dhanakumar et al. [32]
Castilseras, Spain	-	59.4	14.3	43.3	43.0	105.9	Garcia-Orcidales et al. [77]
Hoedong, Korea	1.6	28.7	57.6	17.2	60.5	247.8	Lee et al. [78]
East Dongting Lake, China	2.7	33.1	46.4	30.8	38.2	154.6	Makokha et al. [79]
Honghu Lake, China	0.1	25.2	78.9	23.8	20.7	145.5	Makokha et al. [79]
Mangla Lake, Pakistan	84.5	32.1	25.0	79.7	82.6	9.5	Saleem et al. [80]
Brody Iłżeckiem, Poland	2.5	41.4	16.6	14.3	69.2	345.0	Smal et al. [81]
Zalew Zemborzycki, Poland	0.5	5.9	7.1	6.1	54.2	43.1	Smal et al. [81]
Stare Miasto, Poland	0.3	0.3	1.7	2.7	3.2	10.9	Sojka et al. [11]
Alkkulam-Veil Lake, India	0.3	183.2	53.8	83.7	59.1	123.5	Swarnalatha et al. [82]
Manwan, China	1.4	54.7	38.9	-	47.1	156.7	Wang et al. [83]
Miyun, China	0.0	21.7	-	26.1	-	19.3	Wu et al. [84]
Biliuhe, China	2.0	95.6	35.3	-	62.9	128.0	Zhu et al. [20]
Dahuofang, China	2.4	116.3	73.0	-	52.5	175.0	Zhu et al. [20]

## Data Availability

Not applicable.

## Supplementary Materials

The following supporting information can be downloaded at: https://www.mdpi.com/article/10.3390/ijerph20010324/s1, Table S1: Parameters of the analyzed dam reservoirs and their catchment parameters.
