# Supplementary material for: Ecological and Health Risk Assessments of Heavy Metals Contained in Sediments of Polish Dam Reservoirs
Source: Int J Environ Res Public Health. 2022 Dec 25;20(1):324. doi: 10.3390/ijerph20010324 (PMC9819632; doi:10.3390/ijerph20010324)
Supplement: Supplementary file 1 [file ijerph-20-00324-s001.zip › ijerph-2085176-supplementary.pdf]

**Table S1.** Parameters of the analyzed dam reservoirs and their catchment parameters.

| No. | Reservoir | River | Longitude (dec deg) | Latitude (dec. deg) | Altitude m asl | Shoreline development (-) | Schindler's ratio (-) | Catchment area (km²) | Urban (%) | Agriculture (%) | Forest (%) | Wetland (%) | Water (%) |
|---|---|---|---|---|---|---|---|---|---|---|---|---|---|
| 1 | Besko | Wisłok | 21.93 | 49.55 | 347.5 | 3.8 | 14.7 | 207 | 0.7 | 28.8 | 69.9 | 0.0 | 0.6 |
| 2 | Kozłowa Góra | Brynica | 18.97 | 50.43 | 277.5 | 1.5 | 12.6 | 194 | 11.0 | 39.5 | 47.0 | 0.0 | 2.5 |
| 3 | Chańcza | Czarna Staszowska | 21.05 | 50.64 | 216.7 | 2.3 | 20.0 | 471 | 3.4 | 47.9 | 48.0 | 0.0 | 0.7 |
| 4 | Klimkówka | Ropa | 21.11 | 49.54 | 478.3 | 2.3 | 5.0 | 214 | 0.9 | 29.2 | 68.7 | 0.0 | 1.3 |
| 5 | Łąka | Przszczynka | 18.87 | 49.97 | 250.2 | 1.7 | 14.2 | 166 | 15.4 | 69.4 | 13.0 | 0.0 | 2.2 |
| 6 | Słup | Nysa Szalona | 16.13 | 51.10 | 173.1 | 1.3 | 10.0 | 383 | 8.0 | 65.3 | 25.9 | 0.0 | 0.8 |
| 7 | Bukówka | Bóbr | 15.94 | 50.71 | 532.9 | 1.4 | 3.5 | 57.54 | 5.5 | 45.7 | 46.6 | 0.0 | 2.2 |
| 8 | Nielisz | Wieprz | 23.01 | 50.78 | 196.6 | 3.1 | 49.9 | 1269 | 4.3 | 60.2 | 34.1 | 0.4 | 0.9 |
| 9 | Sosnówka | Czerwonka | 15.71 | 50.83 | 369.2 | 1.4 | 1.2 | 15 | 6.3 | 29.9 | 54.7 | 0.0 | 9.2 |
| 10 | Rzeszów | Wisłok | 21.99 | 50.01 | 199.5 | 6.0 | 2 462.4 | 2068 | 8.3 | 57.0 | 34.6 | 0.0 | 0.1 |
| 11 | Goczałkowice | Mała Wisła | 18.87 | 49.93 | 254.4 | 1.9 | 3.3 | 523 | 14.1 | 37.2 | 40.9 | 0.4 | 7.5 |
| 12 | Przeczyce | Czarna Przemsza | 19.20 | 50.45 | 285.9 | 2.4 | 14.8 | 301 | 13.2 | 43.2 | 42.3 | 0.0 | 1.3 |
| 13 | Dobczyce | Raba | 20.04 | 49.87 | 269.6 | 3.7 | 5.6 | 765 | 7.1 | 43.7 | 47.9 | 0.0 | 1.3 |
| 14 | Niedów | Witka | 15.00 | 51.04 | 216.5 | 2.5 | 65.3 | 318 | 4.6 | 49.6 | 45.6 | 0.0 | 0.2 |
| 15 | Czorsztyn | Dunajec | 20.25 | 49.45 | 522.9 | 2.7 | 4.9 | 1126 | 7.5 | 43.6 | 47.1 | 0.6 | 1.2 |
| 16 | Solina | San | 22.45 | 49.34 | 445.6 | 7.0 | 2.6 | 1191 | 0.9 | 12.8 | 84.2 | 0.0 | 2.1 |
| 17 | Pierzchały | Pasłęka | 19.85 | 54.26 | 21.7 | 4.4 | 182.5 | 2096 | 2.6 | 57.9 | 36.9 | 0.3 | 2.2 |
| 18 | Rożnów | Dunajec | 20.69 | 49.72 | 262.4 | 3.6 | 30.6 | 4855 | 5.7 | 40.9 | 52.5 | 0.2 | 0.7 |
| 19 | Nysa | Nysa Kłodzka | 17.26 | 50.45 | 193.5 | 3.4 | 26.3 | 3263 | 5.6 | 52.9 | 40.2 | 0.1 | 1.2 |
| 20 | Sulejów | Pilica | 19.93 | 51.43 | 165.4 | 4.2 | 58.8 | 4933 | 4.5 | 54.3 | 40.0 | 0.2 | 0.9 |
| 21 | Jeziorsko | Warta | 18.70 | 51.83 | 119.7 | 1.9 | 44.6 | 9007 | 8.1 | 60.2 | 30.9 | 0.1 | 0.7 |
| 22 | Poraj | Warta | 19.23 | 50.65 | 276.5 | 2.5 | 15.7 | 390 | 14.9 | 52.2 | 31.1 | 0.0 | 1.8 |
| 23 | Włocławek | Wisła | 19.27 | 52.63 | 56.0 | 3.8 | 420.4 | 171467 | 6.6 | 59.8 | 31.9 | 0.4 | 1.2 |
| 24 | Dębe | Narew | 21.01 | 52.45 | 78.1 | 11.8 | 731.8 | 68974 | 4.0 | 61.2 | 32.2 | 1.1 | 1.6 |
| 25 | Siemianówka | Narew | 23.81 | 52.93 | 142.6 | 1.5 | 14.1 | 1085 | 1.7 | 35.1 | 44.3 | 8.1 | 10.7 |
| 26 | Turawa | Mała Panew | 18.13 | 50.72 | 174.9 | 1.9 | 13.6 | 1419 | 6.2 | 32.3 | 59.9 | 0.1 | 1.5 |
| 27 | Otmuchów | Nysa Kłodzka | 17.11 | 50.47 | 208.2 | 1.5 | 16.6 | 2358 | 5.5 | 53.8 | 39.6 | 0.1 | 1.0 |
| 28 | Międzybrodzie | Soła | 19.21 | 49.78 | 323.2 | 2.1 | 34.5 | 1100 | 9.0 | 30.5 | 59.3 | 0.0 | 1.2 |
